# Application of theories of the policy process in research on consumption of sustainable diets: a systematic review

**DOI:** 10.1186/s12889-022-13717-5

**Published:** 2022-07-13

**Authors:** Celia Green, Gemma Carey, Andrew Joyce

**Affiliations:** 1 Centre for Social Impact, University of New South Wales, 704, Level 7, Science Engineering Building, Sydney, NSW 2052 Australia; 2 Centre for Social Impact, Swinburne University of Technology, John Street, Hawthorn, Victoria 3122 Australia

**Keywords:** Sustainable diets, Policy, Policy process, Policy theory

## Abstract

**Background:**

There is a significant global lack of policy action on consumption of sustainable diets. Application of political science theories such as theories of the policy process can help in understanding policy inaction. Applying these theories could provide a more in-depth understanding of how various influences on the policy process shape decision making for consumption of sustainable diet policy.

**Methods:**

A systematic review to examine application of eight key political science theories of the policy process to research on consumption of sustainable diets was conducted.

**Results:**

The review identified no papers applying a theory of the policy process although 17 papers did mention or discuss influences on the policy process that are common elements within theories of the policy process. Most notably these elements were the influence of coalitions/networks, evidence use, narratives and framing, institutional and political system factors, and the importance of value and belief systems and socio-cultural norms. However, in most papers these influences were not examined in a detailed or in-depth way and often presented as suggestions for lack of policy action without the support of empirical data or application of any theory.

**Conclusions:**

Most research discussing policy inaction on the consumption of sustainable diets fails to utilise political science theories of the policy process, although a small number of papers include mention of or discussion of influences on the policy process. Application of political science theories could provide a more in-depth understanding of how different determinants might shape decision making at various points in the policy process. This could help identify key reasons for policy inaction on the consumption of sustainable diets and suggest possible ways to increase attention and action on the issue from policy decision makers.

**Supplementary Information:**

The online version contains supplementary material available at 10.1186/s12889-022-13717-5.

## Background

### Policy on consumption of sustainable diets

A systematic review of the environmental and health impacts of dietary change in high-income countries concludes there are clear environmental benefits to modifying current dietary practices towards more sustainable choices, and that environmental benefits are largely proportional to the extent by which meat and dairy consumption can be reduced [1]. However, it is clear policy change to influence public behaviour towards consuming more sustainable diets faces significant policy resistance (see for example: [2–8]). Porritt [9] is not exaggerating when he notes, “Policy makers’ attention to...meat eating is as close to zero as it is possible to get” (p.386). Lang et al. [6] and Westhoek et al. [10]  express similar sentiments about the current situation in which interest in and initiatives regarding consumption of sustainable diet policies, and in particular reduction of meat consumption, are practically nonexistent and seen as politically taboo.

A number of possible reasons for this policy resistance have been put forward, with studies citing the complexities related to contemporary food production and consumption as being the major challenge for policy makers in taking action [7]. The difficulties in changing consumer behaviour have also received attention, for example changing food consumption practices will require a shift in consumer behaviour and this has been seen to be “politically delicate” ([6], p.3). As Lang and Barling [6] argue “demanding or even subtly re-framing consumer behaviour change is an anathema to the neo-liberal ethos of consumer choice and sovereignty” (p.3). Both government and non-government organisations may not wish to impinge on individuals’ lifestyle decisions for fear of alienating supporters and being accused of ‘nanny statism’ [11, 12]. Others have suggested that governments are discouraged from taking action by a perceived lack of evidence of the efficacy of intervention strategies [7] and opposition from powerful interest groups such as the food industry or agricultural producers [13, 14].

Alongside these suggestions there have also been some recent attempts in the literature to examine the processes occurring around the creation and implementation of policies in support of consumption of sustainable diets. These studies have largely focused on describing the competing interests involved in the governance of sustainable diets such as between government, civil society, consumers, and private sector stakeholders [6, 11, 14–19]. Other studies have examined the role of non-government organisations in the promotion of sustainable diet policies [12, 20, 21] and the alignment of research priorities to better reflect policy needs [7, 22]. Such studies are useful for identifying some of the determinants that might be influencing decision making in the policy process around sustainable diets. These have included the knowledge, values and beliefs of policy makers [14, 18, 23, 24] and the evidence-policy gap [7, 15, 22, 25]. Power imbalances between stakeholders [16, 25, 26], the processes occurring in policy networks [14, 24] and the influence of vested interests on policy makers [15] have also been identified as factors affecting policy decisions.

While studies which elucidate potential policy determinants have a role to play in contributing to knowledge about why there has been so little policy action on consumption of sustainable diets, they do not provide any in-depth understanding of *how* these determinants might shape decision making at various points in the policy process. For example the powerful influence of food and agricultural industry lobbying on policy makers has often been cited as a key reason for a lack of policy action, and the production and use of evidence highlighted as a significant factor in shaping policy decisions. Yet there is no research which examines critical questions such as *how* and on *whom* do policy actors exert influence in the policy process? And, how do policy actors form successful coalitions to influence policy decisions, and how are policies on consumption of sustainable diets informed by evidence?

### Political science perspectives on consumption of sustainable diets policy

The questions outlined above are not isolated to the area of sustainable diets and indeed in areas such as public health and environmental policy there has been increasing attention on the need to utilise theories from other disciplines, particularly political science, to help conceptualise and make sense of the many interactions and variables involved in complex policy areas [27–31]. As Breton and de Leeuw argue, without robust theoretical grounding, policy failures and successes cannot be satisfactorily explained and “remain all but anecdotal accounts” ([32], p. 88).

Distinct from the “what have we done” and “what should we do” questions of policy evaluation and analysis, policy process research asks questions about the why and how of policy making. For example policy process scholars seek to find out why certain policy issues capture the attention of governments, how coalitions or groups influence policy making, and how different institutional arrangements and history shape policy. As Breton and de Leeuw [32] identify, theories of the policy process need to be able to explain the links between the goals, values, beliefs, and actions of potentially hundreds of policy actors interacting in the policy process which eventuates in specific policy outcomes. These theories look at the conditions under which particular policy events (i.e. decisions on resource allocation and implementation of policy, preferences for particular intervention types, and inclusion or exclusion of different stakeholders) take place and how these factors determine support or otherwise for particular policy positions [32, 33]. As such, multiple theories are needed to describe, explain and highlight “different and sometimes overlapping or nested partitions of the policy process to account for a variety of interactions” ([34], p.5). There are a number of key political science theories that can help guide this analysis.

De Leeuw et al. [35] describe a theory as a “clear and logically interrelated set of propositions, some of them empirically falsifiable, to explain fairly general sets of phenomena” (p.26). Within the field of political science numerous theories of the policy process have been developed [36] with their application ranging from being able to be broadly applied to any situation to in-depth modelling for very specific situations [32]. While there is no universal guide as to which theories of the policy process can be said to be ‘good’ theories, Sabatier [36], an eminent researcher in contemporary political science, has identified theories of the policy process (see Table 1) which reasonably meet a set of minimal conditions:

1. Meet the criteria of a theory (specifies the scope of inquiry, details assumptions, provides a shared vocabulary amongst members of a research team, and clearly defines and relates concepts in the form of principles, testable hypotheses, and propositions).
2. Is indicative of an active research program via recent theoretical development and empirical applications.
3. Has a relatively broad scope that seeks to explain a substantial portion of the policy process

**Table 1:** Elements forming theories of the policy process (Adapted from [37])

Theory		Scope	Level of analysis	Relationships among key concepts
**1**	**Multiple Streams Approach**	Policy choices under condition of ambiguity	System (implicit) with focus on policy actors coupling the streams	Three independent streams that come together during ‘policy windows’ of opportunity which significantly increases the chance of policy adoption
**2**	**Punctuated Equilibrium Theory**	Political system usually in stable state punctuated by periodic major change	System (implicit)	Factors limiting change or generating incrementalism and factors leading to major change
**3**	**Social Construction Framework**	Target populations and policy dynamics	System (implicit)	Social construction of target groups influences policy agenda and policy rationales
**4**	**Advocacy Coalition Framework**	Interactions of advocacy coalitions influencing policy change	Coalitions and policy subsystems	Factors influencing formation of coalitions, policy change and policy learning
**5**	**Narrative Policy Framework**	How narratives influence policy agendas, policy decisions, policy change and public opinions	Micro (individual), Meso (coalitions, institutions) Macro (Societal)	Influence of narratives on public opinion/mood, coalition strategies, policy making and learning
**6**	**Institutional Analysis and Development Framework**	How institutional arrangements are devised to solve collective action problems and outcomes of these arrangements	“Action arena” any social space where individuals interact	At the theory level: Factors and principles that lead to collective action and principles of the governance of common pool resources
**7**	**Diffusion of Innovations**	Policy diffusion and innovation	Venues/states where policy making takes place	Factors in policy diffusion and adoption
**8**	**Policy Feedback Theory**	Influence of policies on politics and subsequent policy making	System (implicit)	How public policies affect the power of groups, the meaning of citizenship, policy agendas and governance structures – all of which impact future policies

For the purpose of this review ‘Theories of the policy process’ will refer to the eight key theories identified by Sabatier as meeting the minimal set of conditions. These will be the theories that are used to guide the systematic review and analyse the current gaps and limitations of current research into consumption of sustainable diets policy.

Given the large body of literature espousing the need for policies to increase consumption of sustainable diets for both health and environmental reasons, there is a clear need to understand why there has been such a significant lack of policy action on this issue at a global scale. Increasing sustainable diet scholars’ knowledge of how policy works could help make their work more relevant and targeted towards addressing policy barriers which could assist with uptake of sustainable diet policies. One way this knowledge could be increased is through the use of theories of the policy process that examine reasons for policy inaction. This study thus aimed to systematically review the literature on consumption of sustainable diets to determine if any research uses policy process theories from political science to explain the lack of policy action and if so what are these explanations?

## Data and methods

The systematic review was undertaken to broadly align with the guidelines laid out in the PRISMA (Preferred Reporting Items for Systematic Reviews and Meta-Analyses) statement [38]. These guidelines were developed to help researchers use systematic methods which reduce bias to enable the production of “reliable findings from which conclusions can be drawn and decisions made” ([39], p.e2).

This systematic review examines the literature on consumption of sustainable diets to ask the question: Do sustainable diet consumption studies draw on or apply any political science theories of the policy process to help explain policy inaction? A systematic electronic search was conducted in June 2021 to identify relevant publications from the following databases: CINAHL, ScienceDirect, Scopus, Web of Science, ProQuest, PubMed, PLoS and JSTOR. The reference lists for included papers were also searched for relevant studies. Search terms for all databases included the following: (climate change OR greenhouse gas OR sustain*) AND (food OR diet* OR nutrition* OR meat) AND (policy OR policies OR govern*). These search terms were chosen to help include papers that linked diets to sustainability and included some mention of policy. The searched fields were keyword, title and abstract where available. Searches were limited to English language studies published until July 2021.

### Inclusion of relevant studies

Titles (and then abstracts where available) were screened for relevance to consumption of sustainable diets policy. Citations were categorized into two groups: i) possibly relevant studies; and ii) excluded studies. The full text of all candidate studies (i.e., possibly relevant studies) was obtained, using a low threshold for inclusion if there was any doubt. These publications were then screened against the inclusion criteria (see Table 2) to determine eligibility. Rather than examining the entirety of the sustainable diet literature (which includes discussion of agricultural production and food security) this review examines only papers specifically focusing on human consumption of sustainable diets. This is for two reasons, firstly the sustainable diet literature is so broad it would be difficult to examine all facets of it in a single review, and secondly changing population behaviour towards consumption of more sustainable diets has been identified as essential for climate change mitigation but an area that has met with significant policy resistance. Papers were thus excluded if they focused only on production side measures of sustainable diets (i.e. discussed policies aimed at agricultural production) and if they were not specific to consumption of sustainable diets (i.e. examined food systems or food security more generally).

**Table 2:** Inclusion and exclusion criteria for the identification of journal papers reporting on the consumption of sustainable diets

**Inclusion criteria** **The article:**	
1. Is in a peer-reviewed journal indexed in CINAHL, SciDirect, Scopus, Webof Science, ProQuest, PubMed, Jstor, PLOS 2. Written in English 3. Published before July 2021 4. Features in abstract, title, or subject headings the search terms: (greenhouse gas OR climate change OR sustain*) AND (nutri* OR diet* OR food OR meat) AND (policy OR policies OR govern*) 5. Has a focus on the consumption of sustainable diets 6. Draws on or applies a theory of the policy process	
**Exclusion criteria** **The article:**	
1. Has a focus on production side on production side measures of sustainable diets i.e. technological advances to mitigate carbon emissions from agricultural production 2. Does not have a specific focus on sustainable diets (i.e. focuses on food systems or food security more generally) 3. Is an opinion piece, editorial, book review or conference proceeding 4. Does not draw on or apply a theory of the policy process	

**Fig. 1:**
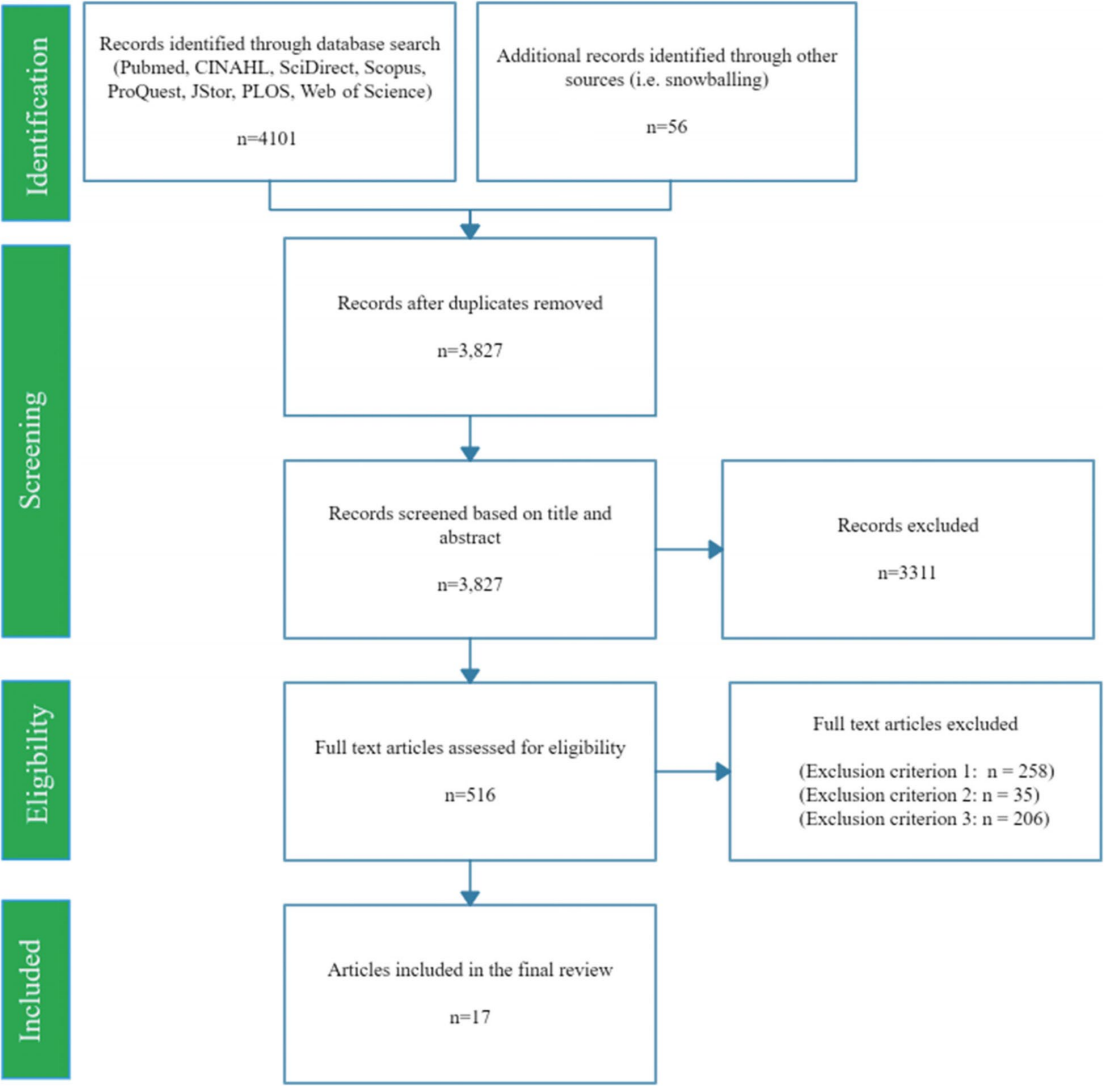
PRISMA flow diagram

Figure 1 outlines the selection process. The search process identified 4157 papers, leaving 3827 after duplicates had been removed. After the titles and/or abstracts were screened for relevance, 516 papers remained. A further 499 papers were excluded after full-text review found that they did not meet the inclusion criteria (258 papers did not focus on consumption of sustainable diets, 35 papers focused on food systems and/or food security more generally rather than a focus on consumption of sustainable diets, 206 papers did not draw on or apply a theory of the policy process). The remaining 17 eligible papers were then further categorised to reflect to what extent they drew on or applied a theory of the policy process to explain a lack of policy action:

1. Applies a theory of the policy process to analyse sustainable diet consumption policy to help explain policy inaction (*N* = 0).
2. Includes mention of or discussion of influences on the policy process consistent with elements from theories of the policy process to help explain policy inaction but does not directly apply a theory of the policy process (*N* = 17).

### Data extraction

Data used for extraction included all text under ‘results’, ‘findings’ or ‘discussion’ sections of the papers. Thus the reviewer took into account all data that appeared to be reporting and discussing results and excluded text that discussed existing literature. A standard data-recording form was used to extract information from included studies. The data extracted was mention of challenges to policy action and/or influences on the policy process. For example whether there was mention of reasons for a lack of policy action on consumption of sustainable diets or factors authors identified as influencing the policy process in this area. These identified ideas or discussion of policy influences were summarised into a table for each of the included studies and from this data six key themes of influence were identified (see Additional file 1: Appendix A for a summary table of key themes extracted from the data).

## Results

### Application of theories of the policy process

This review did not identify any papers applying a key political science theory of the policy process (as identified in Table 1) to help explain policy inaction on sustainable diet consumption policy. However, 17 papers were identified that discussed some of the influences on the policy process that are included elements of political science theories of the policy process (See Table 3). These are examined in the sections below.

**Table 3:** Summary of themes of influence on the policy process by included studies

Author, year	Influences on the policy process					
	Coalitions/ networks	Organisational, institutional and political system factors	Narratives/ Framing	Dominant political ideology	Use of evidence	Personal values, beliefs, and socio-cultural norms
[40]	✓	✓	✓	✓		✓
[41]					✓	✓
[42]	✓	✓				✓
[16]	✓	✓		✓		
[25]	✓	✓	✓		✓	
[43]	✓		✓	✓	✓	✓
[14]	✓	✓			✓	
[24]	✓					
[20]			✓			
[12]	✓				✓	✓
[21]					✓	✓
[44]	✓	✓			✓	
[45]	✓	✓	✓	✓	✓	
[46]	✓	✓				✓
[7, 22]		✓	✓			
[47]	✓					✓
[48]					✓	

### Influence of coalitions/networks

Eleven of the seventeen papers mentioned the influence of coalitions or networks on sustainable diet consumption policy. The way in which coalitions or networks of policy actors are integral parts of the policy process is highlighted in many theories of the policy process, most importantly Kingdon’s Multiple Streams Analysis [49] which discusses the importance of policy entrepreneurs in influencing policy change, and the Advocacy Coalition Framework which organises policy actors into advocacy coalitions based on shared beliefs and co-ordination strategies [50]. While these papers mentioned the importance of coalitions these were not analysed in depth in regard to the strength or otherwise of these networks and only anlaysed in a rudimentary way with regard to how they might influence the policy process.

Eight papers mentioned power imbalances between sustainable diet policy actors [16, 25, 40, 42, 43, 45–47] and identified the food industry and/or farming lobby groups as having greater political access and influence, which was concluded to be a significant reason for limited political action on sustainable diets. However, these eight papers did not conduct extensive analysis of power imbalances, rather they focused on *what* is happening rather than *how* and *why* it is happening. Johnston et al. [14], Joyce et al. [24] and Lawrence et al. [44] mentioned the need to understand policy networks and the way different policy actors influence political agendas with Johnston et al. [14] stating that to overcome political challenges to governance of sustainable diets will require “co-operation, coordination, and negotiation across all stakeholder groups” (p. 426). However without empirical evidence on the values and beliefs of different stakeholders or the ways in which policy actors form coalitions and networks and exert political influence in particular political contexts it is difficult to draw any firm conclusions on their importance as barriers or enablers of policy change.

Two papers by James et al. [25] and Denniss et al. [16] attempted to address this gap by using empirical evidence from food policy actors in Australia to help understand the barriers and enablers of policy action on consumer adoption of healthy and sustainable food behaviours. James et al. [25] conducted semi-structured interviews with food policy stakeholders including the food industry, government, and non-government organisations while Denniss et al. [16] conducted a Delphi study with similar stakeholders. Based on the barriers and enablers identified in the interviews James et al. [25] developed a framework for intersectoral action and Denniss et al. [16] made a number of similar policy recommendations including the need to build relationships with key stakeholders and developing greater understanding of the policy process. These were one of the few examples that provided some more analytic depth on the policy process albeit that a specific political science theory was not used in the analysis. The lack of application of any theories of the policy process to analyse the influence on coalitions and networks to try and explain policy inaction on consumption of sustainable diets highlights the considerable gap in application of political science theories in this area. This means practitioners and policy makers have little research by which to guide their actions and strategies.

### Organisational, institutional and political systems

The study of institutions, organisations and political systems within the policy process has been the focus of much work in political and social sciences [51]. For example the Institutional Analysis and Development Framework is a theory of the policy process which provides a way to think about how different institutions cultivate collective action [52]. Six papers mentioned either organisational factors, institutions, or political systems as impacting sustainable diet policy. From interviews with stakeholders involved in Australian sustainable diet policy James [25] identified that organisations give little attention to sustainable diets as many don’t see it as “core business” largely because of a lack of institutional or political interest or prioritising of sustainable food supply and demand issues. Similarly, in investigating factors influencing NGOs to campaign on reduced meat consumption Laestadius et al. [12, 21] found that depending on their focus (environment, food, health) some NGOs had a low level of engagement with the issue as it was not seen as their primary focus. However, no policy theories were specifically mentioned as having guided the analysis of their empirical data.

Institutions and political systems were discussed by Santaoja and Jauho [46] in the context of the Finnish Dietary Guidelines. They identified a situation of institutional ambiguity in regard to where and who is making sustainable food policy with no political consensus on what sustainable diets are. It was noted that policy makers had little independence in the contexts in which they worked and were thus reluctant to experiment with policy on sustainable diets. Rather than using any policy theories the authors employed the concept of ontonorms to help understand the integration of sustainability into dietary guidelines.

### Narratives/framing

Many policy theories discuss the links between evidence, persuasion, and the framing of policy problems (which sit within a broader environment where particular beliefs may dominant discussions) [53]. One theory of the policy process – the Narrative Policy Framework, explicitly focuses on the importance of narratives in creating policy change [54]. The way sustainable diet policy has been framed was mentioned in five papers [14, 20, 25, 40, 43]. For example Beverland [40] commented that marketers, promoters and critics have often framed vegetarian or “ethical” diets as something for those who are culturally privileged rather than for the mainstream and language around reducing meat consumption is framed in anthropogenic terms which conceals how meat consumption impacts sustainability. Additionally he identified an economic logic as being pervasive in sustainability narratives. Similarly, James et al. [25] showed how in the Australian context economic themes and frames are often used by industry stakeholders when they define sustainability and stakeholders describe concepts of health and sustainability separately, with health receiving the greatest emphasis. In discussing dietary guidelines Jelsøe [43] argued that dietary guidelines act to reproduce and strengthen the discourse around food and health where health issues are seen as isolated from other considerations such as sustainability. However, although some narrative and framing issues were identified as possible barriers to policy action on consumption of sustainable diets no papers utilised any narrative or framing policy theories to analyse sustainable diet narratives in a more in-depth way. Again this is another potential limitation in the literature given the use of a theory to guide the analysis could help reveal possible strategies and actions that increase policy action around consumption of sustainable diets.

### Dominant political ideology

A number of papers made mention of dominant economic or neoliberal political ideology acting as a barrier to policies to encourage consumption of sustainable diets. This often has interactions with other influences on the policy process. For example in their examination of how livestock industry practices influence sustainable diet policy in the US Rose et al. [45] discussed the way industry framed their practices as of economic importance in order to align with the dominant economic rationalist ideology of government. Denniss et al. [16] similarly noted that neoliberal ideology and prioritisation of economic interests act to exacerbate a lack of “political will” by governments to act on sustainable diets. Yet as theories of the policy process show “political will” is merely the end point of a range of inter-related elements coming together to create policy action [55]. This again underscores the way applying theories of the policy process in research on consumption of sustainable diet policy could help elucidate the key factors acting as barriers to policy action.

### Use of evidence

There is a large literature demonstrating the gap between production of evidence and a policy making response [53, 56, 57] in many policy areas including social, environmental and health policy. However studies in these areas have rarely utilised insights from political science policy theories to explain the evidence-policy gap [53, 56]. Similarly although six papers in this review touched on the use of evidence in sustainable diet policy none used a policy theory to further analyse evidence used in particular policy contexts. Dagevos and Voordouw [41] pointed out that despite there being abundant scientific evidence on the sustainability problems of meat consumption “political attention is conspicuously absent”. In contrast James [25] found it was a lack of evidence of what constitutes a sustainable diet that acted as a barrier to inter-sectoral action on sustainable diets. Jelsøe [43], Johnston et al. [14] and Lawrence et al. [44] also commented that there either wasn’t enough evidence on the metrics of sustainable diets in particular contexts or that the evidence was too fragmented or overwhelming for politicians to take meaningful action. Additionally Jelsøe [43] noted that the food industry has questioned the evidence around sustainable diets as a way to avoid sustainability being included in dietary guidelines. Utilising empirical evidence combined with policy theory to explore these hypotheses would enable a greater understanding of barriers to policy action.

### Personal values, beliefs and socio-cultural norms

Theories of the policy process recognise the importance of the public mood, and the values and beliefs of stakeholders in the way the policy process operates. Almost all theories of the policy process include these elements with some such as the Social Construction Framework and Narrative Policy Framework more specifically focusing on these influences. A number of papers in this review commented on the way meat consumption is associated with socio-cultural norms and beliefs. Beverland [40] argued that in developed economies consumption becomes more about expressing identity than fulfilling basic needs with consumption of meat being the most obvious example, reflecting identity issues of gender, class, race, and culture, which acts as a barrier to political action on sustainable diets. Similarly Dagevos & Voordouw [41] commented that it is unlikely consumers would be receptive to policies around reduced meat consumption due to a carnivorous food culture and Jelsøe [43] noted that providing advice on what people should eat can be perceived as a threat to individual choice. In their exploration of NGO messaging Laestadius et al. [12, 21] identified a negative feedback loop on messaging to reduce meat consumption – when governments and the public see the issue as unpopular or uninteresting NGO’s reduce their messaging efforts which in turns deprives the issue of the attention it needs. However none of these papers sought to apply a political science theory to examine in more depth the way these influences could be a significant reason for policy inaction.

## Discussion

This systematic review found no papers that applied a key political science theory of the policy process to help explain policy inaction on consumption of sustainable diet policy. However, a small number of papers did discuss or mention some influences on the policy process that are regularly included as elements within theories of the policy process. Most commonly mentioned was the influence of coalitions or networks on the way sustainable diet consumption policy is being made, with a focus on power imbalances between stakeholders’ ability to influence policy. The power of the food industry and/or farming lobby groups in exerting political influence was mentioned in a number of papers (e.g. [25, 40, 42] and seen as a significant factor in the limited policy action on sustainable diets to date. However, only the papers by James et al. [25] and Denniss et al. [16] used empirical data to investigate this assumption although the authors did not specifically mention having analysed the results by applying a political science theory. Rather barriers and enablers to policy action on sustainable diets were organised thematically with these themes then used to create an action framework designed to help encourage inter-sectoral action [25] and to make policy recommendations [16]. While welcome, this type of research could be further strengthened by applying theories of the policy process. For example using a theory such as the Advocacy Coalition Framework [50] could provide greater insights into the policy core beliefs of sustainable diet advocacy coalitions and how they co-ordinate actions to influence a policy subsystem. The increased insight and knowledge from application of a theory of the policy process would then help strengthen any proposed interventions or solutions such as the action framework devised by James et al. [25].

Other policy influences identified in some of the papers included the narratives and framing used to communicate consumption of sustainable diets as a policy problem, institutional, organisational and political system factors, the use of evidence and the influence of personal values, beliefs and socio-cultural norms. However most papers with the exception of those by James [25], Denniss [16] and Laestadius et al. [12, 20, 21] did not use empirical evidence to support their discussion of these policy influences. Rather different policy influences were presented more as suggestions by the authors to account for the lack of policy action. For example Johnston et al. [14] cite a lack of evidence on measurement of what constitutes a sustainable diet as a reason “policymakers are unable to make decisions or recommendations to advance the concept of sustainable diets” (p.426). While it may be that some of these suggestions are correct, a lack of use of theories of the policy process to test these in a more analytical way limits any conclusions being made about why meaningful political action on consumption of sustainable diets is absent on a global scale. For example application of a theory such as the Narrative Policy Framework [54] which utilises empirical evidence to examine the implicit truth that narratives have power by asking “do narratives play an important role in the policy process?” ([54], p. 225) could provide greater clarity on the narratives in play in sustainable diet policy and how these are shaping policy action/inaction. Likewise examining sustainable diet policy through the lens of a theory such as the Institutional Analysis and Development (IAD) Framework could assist in gaining greater insight into the institutional factors shaping the problem and its potential solutions. The IAD framework originated with a quest by policy scholars to explain how people develop institutional arrangements to “solve collective action problems and provide shared benefits” ([58], p. 271), making it well suited to analysing a complex policy issue like the consumption of sustainable diets.

As identified in a number of papers, personal values, beliefs and socio-cultural norms are also suggested to shape decision making by policy actors and to influence which problems become the focus for policy. For example a few papers in this review discussed the influence of socio-cultural norms on consumption of meat with this being perceived as a barrier to policies on consumption of sustainable diets. Jelsøe [43] hypothesised that socio-cultural meat eating norms reduce politicians willingness to implement policies to shift consumers to eating less meat as they believe these type of policies would be unpopular with the public. Similarly in examining NGO messaging on reducing meat consumption Laestadius found NGO’s reduce their messaging around issues perceived to be unpopular with politicians and the public – creating a negative feedback loop in what politicians perceive to be a problem the public wants addressed. Utilising theories of the policy process can provide increased insight into how values, beliefs and norms influence policy making and how this means certain issues come to be perceived as policy problems decision makers need to solve. Notably Kingdon’s Multiple Streams Analysis [49] includes the “problems stream” which comprises a variety of conditions that citizens and policy makers currently identify as public problems they want addressed [59], such as climate change, reduced health budgets, inflation, and so on. Not all conditions become problems, as Kingdon [55] states, problems contain “a perceptual, interpretive element” (p. 10). Conditions which come to be perceived as problems are the ones which receive the greatest attention. Zahariadis [60] suggests that a range of values is usually associated with a specific issue and that changes in a particular condition may disrupt these values thereby stimulating attention and interest. People also identify conditions as problems by allowing their values and beliefs to guide their decisions. While the MSA has been applied extensively to policies in a variety of settings and across national, sub-national, and supra-national levels, none of the papers in this review utilised this theory to examine consumption of sustainable diets policy.

It should also be noted that the policy change influences identified in papers in this review and which are elements in policy theories are not mutually exclusive and are often highly interrelated [27]. This points to a need for more sustainable diet policy research utilising theories of the policy process that consider a multitude of influences to examining complex policy areas. Theories such as Kingdon’s Multiple Streams Theory, the Advocacy Coalition Framework, and Punctuated Equilibrium Theory for example all consider numerous crucial influences on policy decision making including network and coalition influences, ideas, institutional factors, and the rationality of decision makers as well as the importance of the external socio-cultural and political context. They also highlight how these influences are inter-connected to more clearly explain potential means for policy adoption [34]. Policy scholars have also suggested that gaining a variety of perspectives by employing more than one theory of the policy process can provide a more comprehensive examination of the complexities involved in policy making for a particular policy area [28, 33, 61].

Across the sustainable diet literature as a whole there is a focus on reducing uncertainty for policy makers by presenting more or better evidence. This is evidenced by the largest concentration of papers being diet modelling studies which seek to establish a quantitative evidence base around how different diets contribute to variables such as greenhouse gas emissions, water use, or land use. Almost of these diet modelling studies also make some kind of policy recommendations in their conclusions, commonly around the need for governments to conduct consumer education campaigns or tax carbon intensive foods. However these recommendations are being made without any discussion or regard for factors which influence the policy process or for the socio-cultural or political contexts in which the policies would need to be developed and implemented. As Cairney and Oliver (2017) argue, recognising that policy makers have a tendency to base judgements on their well-established value and belief systems and make decision making shortcuts based on emotions and familiarity with information is a key understanding required for stakeholders advocating policy change. Without a focus on the way policy makers understand and react to problems those seeking to create policy change will find it much harder to exert any influence, and instead find themselves only responding to sudden demands from policy makers for evidence-based solutions to pre-defined problems [62]. Greater utilisation of theories of the policy process to examine policy inaction could help sustainable diet researchers gain a better understanding of how policy making works and where the barriers to policy action lie. This could help them tailor their policy recommendations to better reflect what is achievable and what would be most impactful in specific contexts (i.e. in different political situations, countries, or levels of governance) rather than making recommendations that have very little chance of success.

Given that food consumption is a complex and interconnected policy area that encompasses a vast literature including food security and production issues, a limitation of this review is that it may have missed studies which have used policy theories to examine other food sustainability policy issues. Never the less this review highlights that in the area of consumption of sustainable diets there is the need for greater use of political science theories of the policy process to help analyse the lack of meaningful policy action from governments across the world. This could provide greater clarity on how policy decision making is being made in this area, where there may be increased opportunities for policy advocacy efforts and to identify where barriers to policy adoption lie. Without application of such theories studies may be more limited in their ability to explain a lack of policy action and elucidate which policy options would be most likely to gain the attention of decision makers. Further, a greater understanding of how the policy process is operating for consumption of sustainable diet policy could help inform policy actors on *how* they can best influence future policy making and the best strategies for different political and socio-cultural contexts. Given the ample evidence on the need for a global shift to public consumption of more sustainable diets to help mitigate climate change and other environmental impacts, future research that applies political science theories of the policy process to help understand policy inaction is urgently needed.

## Conclusion

This review examined the application of theories of the policy process to research on consumption of sustainable diets to help explain policy inaction. No papers were found that directly applied a theory of the policy process although a small number of papers were identified that did include some discussion of key influences on policy making that are commonly included elements within theories of the policy process. However, these were often only examined in a rudimentary way and most papers lacked empirical evidence to support suggestions for policy inaction. A lack of application of theories of the policy process means studies are more limited in their ability to explain the absence of policy action or to recommend strategies and leverage points which would help achieve future policy change. Given the urgent need to shift consumers towards more sustainable diets to help mitigate climate change and other environmental impacts we recommend more research utilising theories of the policy process to help explain this lack of policy action and provide greater insight into how to achieve increased attention and policy implementation by decision makers.

## Supplementary Information

**Additional file 1: Appendix A.** Summary of key themes of policy influence extracted from the data.

## Data Availability

Not applicable.

## References

[1] Aleksandrowicz L, Green R, Joy EJM, Smith P, Haines A (2016). The impacts of dietary change on greenhouse gas emissions, land use, water use, and health: a systematic review. PLoS One.

[2] Bailey R, Froggatt A, Wellesley L (2014). Livestock—climate change’s forgotten sector: global public opinion on meat and dairy consumption.

[3] Bristow E, Fitzgerald A (2011). Global climate change and the industrial animal agriculture link: the construction of risk. Soc Anim.

[4] Deckers J (2013). Obesity, public health, and the consumption of animal products. Journal of Bioethical Inquiry.

[5] Garnett T, Mathewson S, Angelides P, Borthwick F (2015). Policies and actions to shift eating patterns: what works? Food Climate Research Network and Chatham House.

[6] Lang T, Barling D (2013). Nutrition and sustainability: an emerging food policy discourse. Proc Nutr Soc.

[7] Sedlacko M, Reisch L, Scholl G (2013). Sustainable food consumption: when evidence-based policy making meets policy-minded research—introduction to the special issue. Sustain Sci Pract Policy.

[8] Vázquez-Rowe I, Larrea-Gallegos G, Villanueva-Rey P, Gilardino A (2017). Climate change mitigation opportunities based on carbon footprint estimates of dietary patterns in Peru. PLoS One.

[9] Porritt J (2010). When evidence-based synergies remain ignored. Health Educ Res..

[10] Westhoek H, Lesschen JP, Rood T, Wagner S, De Marco A, Murphy-Bokern D (2014). Food choices, health and environment: effects of cutting Europe’s meat and dairy intake. Glob Environ Change..

[11] Dixon J, Isaacs B (2013). Why sustainable and ‘nutritionally correct’ food is not on the agenda: Western Sydney, the moral arts of everyday life and public policy. Food Policy.

[12] Laestadius LI, Neff RA, Barry CL, Frattaroli S (2014). “We don’t tell people what to do”: an examination of the factors influencing NGO decisions to campaign for reduced meat consumption in light of climate change. Glob Environ Chang.

[13] Garnett T (2014). Changing what we eat: a call for research & action on widespread adoption of sustainable healthy eating. Food Climate Research Network.

[14] Johnston JL, Fanzo JC, Cogill B (2014). Understanding sustainable diets: a descriptive analysis of the determinants and processes that influence diets and their impact on health, food security, and environmental sustainability. Adv Nutr.

[15] Reisch LA, Eberle U, Lorek S (2013). Sustainable food consumption: an overview of contemporary issues and policies. Sustain Sci Pract Policy.

[16] Denniss E, Woods J, Lawrence M (2021). Promoting healthy and sustainable diets: barriers and enablers for successful policy activities in Australia.

[17] Ridgway EM, Lawrence MA, Woods J. Integrating environmental sustainability considerations into food and nutrition policies: insights from Australia’s National Food Plan. Front Nutr. 2015;2. 10.3389/fnut.2015.00029. 10.3389/fnut.2015.00029 PMC4585016 26442275

[18] Smith J, Lang T, Vorley B, Barling D (2016). Addressing Policy Challenges for More Sustainable Local–Global Food Chains: Policy Frameworks and Possible Food “Futures”. Sustainability.

[19] Sulda H, Coveney J, Bentley M (2010). An investigation of the ways in which public health nutrition policy and practices can address climate change. Public Health Nutr.

[20] Laestadius LI, Neff RA, Barry CL, Frattaroli S (2013). Meat consumption and climate change: the role of non-governmental organizations. Clim Chang.

[21] Laestadius LI, Neff RA, Barry CL, Frattaroli S (2014). No meat, less meat, or better meat: understanding NGO messaging choices intended to Alter meat consumption in light of climate change. Environ Commun.

[22] Sedlacko M, Pisano U, Berger G, Lepuschitz K (2013). Bridging the science-policy gap: development and reception of a joint research agenda on sustainable food consumption. Sustain Sci Pract Policy.

[23] Garnett T (2013). Food sustainability: problems, perspectives and solutions. Proc Nutr Soc.

[24] Joyce A, Hallett J, Hannelly T, Carey G. The impact of nutritional choices on global warming and policy implications: examining the link between dietary choices and greenhouse gas emissions. Energy Emission Control Technol. 2014;33. 10.2147/EECT.S58518.

[25] James S, Friel S, Lawrence MA, Hoek AC, Pearson D (2018). Inter-sectoral action to support healthy and environmentally sustainable food behaviours: a study of sectoral knowledge, governance and implementation opportunities. Sustain Sci.

[26] Lerner H, Algers B, Gunnarsson S, Nordgren A (2013). Stakeholders on meat production, meat consumption and mitigation of climate change: Sweden as a case. J Agric Environ Ethics.

[27] Clarke B, Swinburn B, Sacks G (2016). The application of theories of the policy process to obesity prevention: a systematic review and meta-synthesis. BMC Public Health.

[28] Clavier C, de Leeuw E (2013). Health promotion and the policy process.

[29] Embrett MG, Randall GE (2014). Social determinants of health and health equity policy research: exploring the use, misuse, and nonuse of policy analysis theory. Soc Sci Med.

[30] Fafard P (2015). Beyond the usual suspects: using political science to enhance public health policy making. J Epidemiol Community Health.

[31] Rickards L, Wiseman J, Kashima Y (2014). Barriers to effective climate change mitigation: the case of senior government and business decision makers. Wiley Interdiscip Rev Clim Chang.

[32] Breton E, de Leeuw E (2011). Theories of the policy process in health promotion research: a review. Health Promot Int.

[33] Breton E, Richard L, Gagnon F, Jacques M, Bergeron P (2008). Health promotion research and practice require sound policy analysis models: the case of Quebec’s tobacco act. Soc Sci Med.

[34] Weible CM, Sabatier PA, Weible CM (2014). Introducing the scope and focus of policy process research and theory. Theories of the policy process.

[35] de Leeuw E, de, & Breton, E., Clavier C, de Leeuw E (2013). Policy change theories in health promotion research: a review. Health promotion and the policy process.

[36] Sabatier PA (2007). Theories of the policy process, second edition.

[37] Cairney P, Heikkila T, Sabatier PA, Weible CM (2014). A comparison of theories of the policy process. Theories of the policy process.

[38] Moher D, Liberati A, Tetzlaff J, Altman DG, Group, T. P (2009). Preferred reporting items for systematic reviews and Meta-analyses: the PRISMA statement. PLoS Med.

[39] Liberati, A., Altman, D. G., Tetzlaff, J., Mulrow, C., Gøtzsche, P. C., Ioannidis, J. P. A., Clarke, M., Devereaux, P. J., Kleijnen, J., & Moher, D. (2009). The PRISMA statement for reporting systematic reviews and meta-analyses of studies that evaluate health care interventions: explanation and elaboration. J Clin Epidemiol Elmsford, 62(10), e1-34. http://dx.doi.org.ezproxy.lib.swin.edu.au/10.1016/j.jclinepi.2009.06.006 10.1016/j.jclinepi.2009.06.006 19631507

[40] Beverland MB. Sustainable eating: mainstreaming plant-based diets in developed economies. J Macromark. 2014:7–10. 10.1177/0276146714526410.

[41] Dagevos H, Voordouw J (2013). Sustainability and meat consumption: is reduction realistic. Sustain Sci Pract Policy.

[42] de Bakker E, Dagevos H (2012). Reducing meat consumption in Today’s consumer society: questioning the citizen-consumer gap. J Agric Environ Ethics.

[43] Jelsøe E (2015). Dietary guidelines: nutritional health communication versus sustainable food policy. J Transdiscipl Environ Stud.

[44] Lawrence MA, Friel S, Wingrove K, James SW, Candy S (2015). Formulating policy activities to promote healthy and sustainable diets. Public Health Nutr.

[45] Rose D, Vance C, Lopez MA (2021). Livestock industry practices that impact sustainable diets in the United States. Int J Sociol Agric Food.

[46] Santaoja M, Jauho M (2020). Institutional ambiguity and ontological politics in integrating sustainability into Finnish dietary guidelines. Sustainability.

[47] Seed B (2015). Sustainability in the Qatar national dietary guidelines, among the first to incorporate sustainability principles. Public Health Nutr.

[48] Simmonds P, Vallgårda S (2021). “It’s not as simple as something like sugar”: values and conflict in the UK meat tax debate. Int J Health Governance.

[49] Kingdon J (1995). Agendas, alternatives, and public policies.

[50] Jenkins-Smith HC, Nohrstedt D, Weible CM, Sabatier PA (2014). The advocacy coalition framework: foundations, evolution, and ongoing research. Theories of the policy process.

[51] Mahoney J, Thelan K (2010). Explaining institutional change: ambiguity, Agency and Power.

[52] Cairney P (2019). Policy concepts in 1000 words: the institutional analysis and development framework (IAD) and governing the commons. Paul Cairney: Politics & Public Policy.

[53] Cairney P, Oliver K, Wellstead A (2016). To bridge the divide between evidence and policy: reduce ambiguity as much as uncertainty. Public Adm Rev.

[54] McBeth M, Jones M, Shanahan E, Sabatier PA, Weible CM (2014). The narrative policy framework. Theories of the policy process.

[55] Kingdon JW (1995). Agendas, alternatives, and public policies.

[56] Clavier C, de Leeuw E, Clavier C, de Leeuw E (2013). Framing public policy in health promotion: ubiquitous, yet elusive. Health promotion and the policy process.

[57] VanLandingham G, Silloway T (2016). Bridging the gap between evidence and policy makers: a case study of the pew-MacArthur results first initiative. Public Adm Rev.

[58] Ostrom E, Cox M, Schlager E, Sabatier PA, Weible CM (2014). An assessment of the institutional analysis and development framework and introduction of the social-ecological systems framework. Theories of the policy process.

[59] Henstra D (2010). Explaining local policy choices: a multiple streams analysis of municipal emergency management. Can Public Admin.

[60] Zahariadis N, Sabatier PA, Weible CM (2014). Ambiguity and multiple streams. Theories of the policy process.

[61] Nowlin MC (2011). Theories of the policy process: state of the research and emerging trends. Policy Stud J.

[62] Cairney P (2011). Understanding public policy: theories and issues (2011 edition).

