# Supplementary material for: Application of theories of the policy process in research on consumption of sustainable diets: a systematic review
Source: BMC Public Health. 2022 Jul 13;22:1335. doi: 10.1186/s12889-022-13717-5 (PMC9281071; doi:10.1186/s12889-022-13717-5)
Supplement: Supplementary file 1 — Additional file 1: Appendix A. Summary of key themes of policy influence extracted from the data. [file 12889_2022_13717_MOESM1_ESM.docx]

**Appendix A**

**Summary of key themes of policy influence extracted from the data**

| **Author, year** | **Main theme of paper** | **Policy process influences** | | | | | | **Policy recommendations** |
| --- | --- | --- | --- | --- | --- | --- | --- | --- |
|  |  | **Influence of coalitions/networks** | **Organisational, institutional and political system factors** | **Narrative and framing** | **Dominant political ideology** | **Use of evidence** | **Personal values, beliefs & socio-cultural norms** |  |
| Beverland, 2014 | Reducing meat consumption | Industry and farming interests linked with scientists who receive funding from them, politicians accepting donations, committees setting dietary guidelines.  Consumers influenced by marketing from food providers. | Governments protect meat industry due to export contribution to economy. | Language around reducing meat consumption framed in anthropogenic terms which obscures impact of meat consumption on sustainability for consumers.  Marketers, promoters and critics frame sustainable diets as something for culturally privileged | Economic logic central to many sustainability narratives – could be used for case of plant-based diets. |  | Consumption in developed economies is more about expressing identity than fulfilling basic needs –most obvious in consumption of meat which reflects identity issues such as class, race, gender, culture | Information provision for consumers.  Use high profile chefs, policy makers or influencers to promote dietary advice  Use of meat analogues or lab meat to provide pathway to plant-based diets  Social support for young and male consumers i.e. via positive role models such as sporting figures.  Further research into message framing for successful marketing campaigns. |
| Dagevos & Voordouw, 2013 | Reducing meat consumption |  |  |  |  | Despite ample scientific evidence of ecological problems of meat consumption political attention is conspicuously absent. | Consumer receptivity to government policies to ban or significantly reduce meat consumption is unlikely due to carnivorous food culture | European member states could pursue consumer oriented policies that encourage reduction in meat consumption without endorsing drastic cuts or bans.  Practical reality of current European policy making should be explicitly taken into account – thus incremental policy strategy is required such as using the four E’s policy framework of series of governance interventions – enabling & encouraging (focused on changing institutional and structural conditions influencing consumers food choices i.e. reorganising provisioning infrastructures and price signals) and exemplifying & engaging (directed to socio-cultural conditions i.e information campaigns, and governments & public bodies acting as role models and market makers around plant based diets). |
| de Bakker & Dagevos, 2012 | Reducing meat consumption | Consumers as agents of change towards reduced meat consumption.  Although sustainable food choices may be blocked by institutional conditions shaping shopping environment (i.e. economic interests of big food companies & marketing strategies) this underestimates the critical capacities of consumers as active agents of change towards reduced meat consumption. | Governments have responsibility to create systems that make it easier for consumers to respond to emerging norm of acting in an environmentally sustainable way. |  |  |  | Changing socio-cultural environment (i.e. advertising, descriptive norms, cultural embeddedness & symbolic meaning of meat eating) will be a lengthy process. | Consumers can be agents of change and should be considered allies trusted with challenge of realisation that reduced meat consumption will contribute to a more sustainable food system. |
| Denniss et al., 2021 | Policy | Competing interests of stakeholders and pressure from the food industry is a barrier to policy action on healthy sustainable diet policy in Australia | Government silos, focus on economism, short term election cycles & lack of political will has an inhibitory effect on sustainable diet policy formation and implementation |  | Neoliberal ideology and prioritisation of economic interests exacerbates lack of political will to adopt sustainable diet policies. |  |  |  |
| (James et al., 2018) | Health | Dysfunctional relationships between food system actors with power imbalances (food industry has greater political access & influence) and mistrust between groups (industry, civil society, consumers).  Government needs to give more support and access to non-government groups to be part of the policy process. Accountability measures must be incorporated into such processes. | Organisations lack attention to healthy and sustainable food behaviours as many don’t see it as “core business”.  One of the  main reasons given for this was a lack of political or  institutional interest in, or prioritising of, healthy and sustainable  supply and demand food issues. As one NGO  representative argued ‘the current [Federal] government  doesn’t have any interest as far as I can see in environmental  anything, and that is definitely a barrier (NGO2)’.  The perspective of other government, NGO and industry  representatives’ was that there were so many issues competing  for attention; it was difficult for healthy and sustainable  food supply and demand issues to get ‘airspace’ at  the organisational level (G5). | Concepts of health and sustainability described separately by stakeholders, with health emphasised over sustainability apart from small number that had integrated definitions.  Economic themes often used by industry stakeholders in definitions of sustainability. |  | Lack of evidence on what constitutes healthy and sustainable food behaviours identified as barrier to inter-sectoral action – with concerns regarding potential unintended consequences of action. |  | Action framework to guide development and implementation of different governance modes and policy, informed by inter-sectoral collaboration between different policy actors. |
| Jelsøe, 2015 | Dietary guidelines | Intense lobbying from food industry & farmers associations prevents sustainability being included in DGs. |  | DG’s contribute to reproducing & strengthening a discourse on food & health where food related health concerns are isolated from other considerations. | Tendency of conservative governments to shelve proposals on environmentally related DGs. | Contrast between expert views and those of ordinary lay people which is inherent in official health communication.  Conflicts in politics of knowledge – expressed through differentiation of scientific expertise & splitting of food science into different disciplines.  Food industry has questioned the underlying evidence for proposals to include sustainability in DGs. | Advice on eating can be perceived as a threat to individual choice. | Board policy processes of dialogue & stakeholder involvement needed.  Transparency on how DGs have been shaped (i.e. role of evidence vs interest groups).  Need an open reflexive approach & understanding of discursive nature of DGs as distinct from focus on “sound science”. |
| Johnston et al., 2014 | Policy | Competing interests of government, civil society, consumers, & private sector present challenges for advancing & operationalising SDs – need to consider preferences & social welfare trade-offs that policy makers & consumers are prepared to make. | Government agencies (agriculture, food, health) work separately with little interaction & may have distinct & contradictory objectives. |  |  | Policy makers not well positioned to take meaningful action to promote SDs because they are not equipped with knowledge or tools to work on the issue – lack of SD metrics, information & data systems |  | Need to develop metrics & measurements for SDs so policymakers can understand how they might improve health & environment & understand trade offs for promoting SDs.  Multisector analysis, cooperation, coordination & negotiation required across all stakeholder groups for political action to occur. |
| Joyce et al., 2014 | Policy | Need to understand networks (consisting of individuals, coalitions, organisations) in policy making to understand processes through which different actors shape and re-shape political agendas. |  |  |  |  |  | More research on message framing to the public which in turn will apply political pressure. Need to understand how evidence can be translated to policy that will suit particular government ideologies. Evidence alone insufficient for driving policy change – need more understanding of political and policy contexts. |
| Laestadius et al., 2013 | Reducing meat consumption |  |  | Limited message promotion on reducing meat consumption from NGOs especially from environmental NGOs.  Lack of message consistency between NGOs. |  |  |  | Continued need for further campaigns with clear messages and well as efforts to build public support for policy measures aimed at reducing meat consumption. |
| Laestadius et al., 2014 | Reducing meat consumption | Low level of engagement with issue of reduced meat consumption especially in environmental NGOs.  Some NGOs reluctant to campaign on issue due to belief associated message on meat and climate change may further agricultural lobby efforts to promote intensive farming options. |  |  |  | Recognition of evidence on need to reduce meat consumption for sustainability not sufficient to motivate NGOs to campaign on the issue.  When NGOs did significantly campaign it was due to secondary benefits to actual core mission rather than evidence compelling them to act. | NGOs face negative feedback loop on reduced meat consumption and climate change – when issue is seen as unpopular/uninteresting by governments and public NGO’s reduce efforts – in turn depriving issue of attention it needs. | Need for more environmental NGOs to adopt campaigns on reduced meat consumption potentially in partnership with animal protection or food focused NGOs. In the absence of this may need expansion of current, or new, food focused NGOs that have narrowly defined missions focusing on food consumption and environment to apply public and political pressure. |
| Laestadius et al., 2014b | Reducing meat consumption |  |  |  |  |  | Factors NGOs consider when developing message choices consistent across nations and NGO types.  Meat consumption plays significant and cultural roles across different nations. Public perceptions on meat consumption rather than climate change currently deciding factor on NGOs messaging decisions. | Need to engage with NGO communications staff to better understand their perception of short-term vs long-term trade off on environmental messaging. |
| Lawrence et al., 2015 | Policy | Need to examine & change current power structures and relationships among actors in the system  Change to the way stakeholders participate in sustainable diet policy making is required | Governance arrangements need to change from individual department projects to whole of government approach |  |  | Can be lack of context on collected data & uncertainty about usefulness of data for policy making.  Large amount of evidence can be overwhelming for policy makers – results in food policy activities being fragmented & politicised. |  | To help shift policy conceptual frameworks can assit in orienting multiple interconnected causes & consequences of problems. Schema highlighting political realties in policy making can help operationalise the framework concepts to policy action to re-design the food system. These in combination provide a policy formulation tool. |
| Rose et al., 2021 | Meat reduction | Corporate political activities of the livestock industry jeopardize progress on sustainable diet policy in the US. | Livestock industry able to exert influence on policy via donations & financial inducements to policymakers | Livestock industry frames the debate by stressing good traits of food industry and exaggerating opponents arguments. | Economic importance of livestock industry made dominant | Livestock industry shapes the evidence base (pay scientists as advisors, supress dissemination of research, fund academics, criticise evidence, cherry pick data, criticise authority of scientists) |  |  |
| Santaoja & Jauho, 2020 | Dietary guidelines | Ontological politics occurs i.e. Finland’s National Nutrition Council aims to be independent but is forced into role of policy maker negotiating with other stakeholders (i.e. food industry). | Institutional ambiguity in food policy as highlighted by dietary guidelines in Finland.  No political consensus on what is a sustainable diet and the issue is “thoroughly political”. |  |  |  | Growing acceptance of governance on sustainable consumption – suggest guidelines on sustainability with clear choice architecture could be widely accepted. |  |
| Sedlacko et al., 2013 | Policy |  | Policy makers have limited independence within their contexts and thus a reluctance to experiment.  Sustainable food consumption as a policy area is not yet fully institutionalised in most EU states & lacks strategic integrated approach. | Knowledge brokerage has challenge of establishing a link to the framing & issues as there is currently no established sustainable food consumption policy base. |  |  |  | For knowledge transfer - When dealing with bureaucratic context the process should try and match the complexity that happens due to functional fragmentation of knowledge. |
| Seed, 2015 | Dietary guidelines | Relative to other larger countries stakeholder involvement in development of dietary guidelines was more limited – with process being more “top down”. This contributed to more rapid development and facilitated integration of sustainability.  Lack of food industry influence on guidelines contributed to inclusion of sustainability. |  |  |  |  | Islamic law provided a connection point for individuals on issues of overconsumption and water. | Population needs to be educated on importance and relevance of the dietary guideline principles.  Champions in local & national sustainability groups could assist with public awareness. |
| Simmonds & Vallgårda, 2021 | Meat reduction |  |  |  |  | Arguments on meat-taxation align with different political ideologies and perspectives on food sustainability. |  |  |
